# An Ecohydraulic Model to Identify and Monitor Moapa Dace Habitat

**DOI:** 10.1371/journal.pone.0055551

**Published:** 2013-02-07

**Authors:** James R. Hatten, Thomas R. Batt, Gary G. Scoppettone, Christopher J. Dixon

**Affiliations:** 1 U.S. Geological Survey, Western Fisheries Research Center, Columbia River Research Laboratory, Cook, Washington, United States of America; 2 U.S. Geological Survey, Western Fisheries Research Center, Reno Field Station, Reno, Nevada, United States of America; Pacific Northwest National Laboratory, United States of America

## Abstract

Moapa dace (*Moapa coriacea*) is a critically endangered thermophilic minnow native to the Muddy River ecosystem in southeastern Nevada, USA. Restricted to temperatures between 26.0 and 32.0°C, these fish are constrained to the upper two km of the Muddy River and several small tributaries fed by warm springs. Habitat alterations, nonnative species invasion, and water withdrawals during the 20th century resulted in a drastic decline in the dace population and in 1979 the Moapa Valley National Wildlife Refuge (Refuge) was created to protect them. The goal of our study was to determine the potential effects of reduced surface flows that might result from groundwater pumping or water diversions on Moapa dace habitat inside the Refuge. We accomplished our goal in several steps. First, we conducted snorkel surveys to determine the locations of Moapa dace on three warm-spring tributaries of the Muddy River. Second, we conducted hydraulic simulations over a range of flows with a two-dimensional hydrodynamic model. Third, we developed a set of Moapa dace habitat models with logistic regression and a geographic information system. Fourth, we estimated Moapa dace habitat over a range of flows (plus or minus 30% of base flow). Our spatially explicit habitat models achieved classification accuracies between 85% and 91%, depending on the snorkel survey and creek. Water depth was the most significant covariate in our models, followed by substrate, Froude number, velocity, and water temperature. Hydraulic simulations showed 2–11% gains in dace habitat when flows were increased by 30%, and 8–32% losses when flows were reduced by 30%. To ensure the health and survival of Moapa dace and the Muddy River ecosystem, groundwater and surface-water withdrawals and diversions need to be carefully monitored, while fully implementing a proactive conservation strategy.

## Introduction

Anthropogenic factors negatively affect aquatic communities in the southwestern U.S. Specifically, in the Southern Xeric Basin and Range ecoregion [1], 82% of sampled stream reaches have disturbed riparian zones, 73% contain non-native vertebrates, 53% have serious streambed stability issues, 42% have mercury in fish, and 33% have reduced habitat complexity [2]. Aggravating this situation is the higher than average human growth rate in the arid southwest, contributing to the 15–60 m declines in groundwater levels region-wide, depending on location [3]. Thus it is no surprise that the desert southwest has an inordinate number of federally listed fishes, including Moapa dace *Moapa coriacea* [4]. Further complicating this picture is the looming threat of climate change, which will likely result in warmer air and water temperatures, reduced winter snowpack, and lower summer streamflows [5], [6]. Collectively, these conditions make it imperative that wise water management practices are implemented to conserve the native aquatic biota in the arid southwest.

The Moapa dace is a thermophilic minnow endemic to the Muddy River, Clark County, Nevada [7]. Inhabiting water temperatures between 26.0 and 32.0°C, Moapa dace is restricted to the upper reaches of the Muddy River ecosystem where the river originates from thermal springs emanating from a deep carbonaceous aquifer [8], [9]. The Moapa dace occurs only in the upper reaches of the Muddy River ecosystem (a.k.a. Warm Springs Area) because its water cools in a downstream direction [10]. In addition, seven other aquatic species of special concern inhabit the Muddy River ecosystem (three fish, two snails, and two insects), with each species having a unique life history and habitat preferences [11]. The Moapa White River springfish *Crenichthys baileyi moapae* is a cohabitating endemic thermophile that occurs in similar locations as Moapa dace. Virgin River chub *Gila seminuda* were known to occur throughout the main stem Muddy River, while speckled dace *Rhinichthys osculus moapae* inhabited the river downstream of the Warm Springs Area.

Moapa dace habitat was altered with the development of spring discharge in the Warm Springs Area for agricultural and recreational use [11], [12]. The introduction of western mosquitofish *Gambusia affinis* by the 1930s and shortfin molly *Poecilia mexicana* in the 1960s also contributed to Moapa dace decline [13], [14]. To insure persistence of Moapa dace and the Moapa White River springfish, the Moapa Valley National Wildlife Refuge (hereafter “Refuge”) was established in 1979 and subsequently expanded [11]. The Refuge is now comprised of three spring provinces (i.e., groups of springs) representing less than 10% of the two endemic’s historic habitat. Still, the Refuge has been important to native fish persistence, especially Moapa dace and White River springfish. Moapa dace reproduce in the spring-fed tributaries to the Muddy River in water temperatures between 30 and 32°C [12].

The Refuge was instrumental in averting the extinction of Moapa dace after the 1995 invasion of blue tilapia *Oreochromis aureus* into the Warm Springs Area. Following the invasion, the two thermal endemic species were extirpated from about 90% of their former range [15], [16], including critical adult foraging habitat in the mainstem Muddy River. While tilapias were prevented from accessing the Refuge by installation of temporary barriers, they have nonetheless temporarily severed the connectivity between springbrook and mainstem habitats. Readers may view a video of Moapa dace and Moapa White River springfish foraging and feeding in the Refuge (see Video S1).

Repatriation of Moapa dace to its historic range (i.e., Muddy River) is important because fragmented populations have a much greater chance of extinction [17], [18]. The largest, oldest, and most fecund Moapa dace occurred in the larger water volume of the main stem Muddy River [12] - life history traits which enhance the species’ probability of persistence [19]. In 2005 the primary water purveyor for Clark County, Southern Nevada Water Authority [20], purchased the Warm Springs Area for the protection of the area’s biota, which provided the opportunity for tilapia extirpation from the Warm Springs Area.

With the establishment of Refuge and the Warm Springs Natural Area (WSNA), a substantial portion of the Moapa dace historic habitat is now under protection. However, in recent years there has been concern as to the sustainability of springs feeding the Muddy River [21]. Specifically, there has been pumping from the Muddy River’s ground-water source, which may increase further, translating into decreased spring discharge [21]. To manage Moapa dace populations on the Refuge and WSNA, while sustaining the seven other sensitive aquatic species, managers need to understand the effect reduced streamflow has on the dace population and the larger Muddy River ecosystem.

In this paper we examine the potential effects of surface-water reductions on the availability of Moapa dace habitat by simulating an increase or decrease in the three primary Refuge springbrooks by 30% relative to baseflow. While Moapa dace are more sensitive to flow reduction than some species (e.g., Moapa White River springfish) [14], our results have implications for all aquatic species in the Warm Springs and Muddy River ecosystem. By providing a methodology that couples fine-grain hydrodynamic data, GIS, and habitat use observations, our approach can be applied to any aquatic ecosystem, large or small, provided the necessary physical and biological data are available.

## Materials and Methods

### Study Site

**Figure 1:**
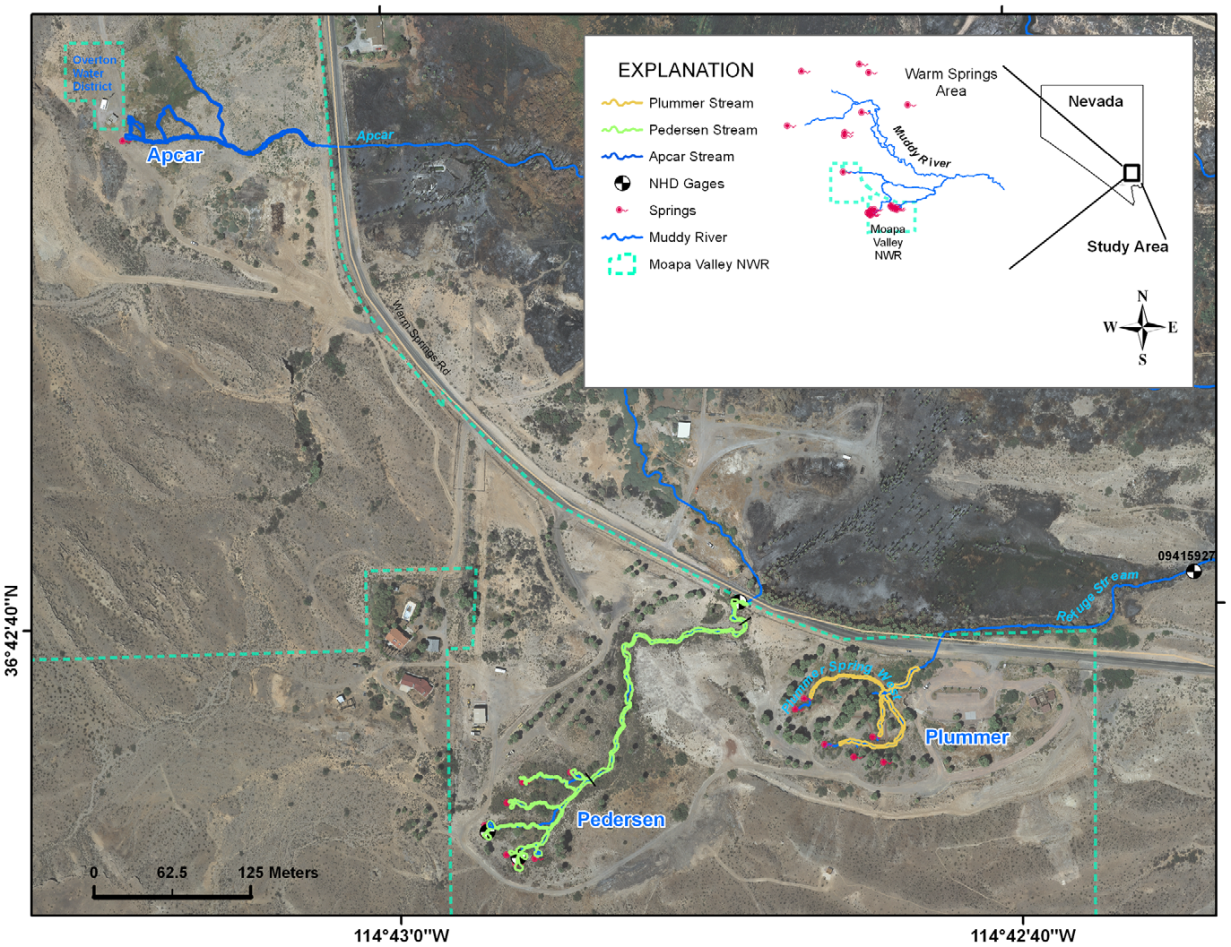
A map of the project area with the three spring-fed creeks displayed inside the Refuge boundary. Culverts route the springbrooks under the road located on the Refuge boundary.

The Moapa Valley National Wildlife Refuge is situated near the southern edge of the Warm Springs Area (Fig. 1). Approximately 47 hectares, the Refuge contains three spring provinces - each of which feed a springbrook - referred to herein as the Plummer, Pedersen, and Apcar springbrooks. The three springbrooks eventually converge to form the Refuge Springbrook, a tributary to the Muddy River. Just prior to their acquisition, the Plummer and Pedersen properties were public resorts with their springbrooks feeding large and small swimming pools. In contrast, Apcar Springbrook had been altered to provide water for local municipal and irrigation purposes. At the time of each acquisition, no Moapa dace and few to no Moapa White River springfish occurred on each of the three properties. Following acquisition by the U.S. Fish and Wildlife Service, substantial habitat rehabilitation was undertaken at each of the three spring provinces aimed at creating suitable native fish habitat. Major rehabilitation modifications included channel realignment, removal of hundreds of nonnative fan palms *Wahingtonia filifera*, and channel excavation. Other rehabilitation actions included riparian vegetation planting, in-stream log placement, and cattail *Tyha* sp. removal [20].

The Pedersen Springbrook system was the first U.S. Fish and Wildlife Service acquisition (1979 and 1984), and habitat modification on that system began in the mid-1980s. This springbrook is fed by the highest springs within the Warm Springs Area and they are suspected to be the most sensitive to ground-water pumping [21]. Of the seven springs feeding the Pedersen Springbrook, two of the highest are equipped with flow gages, as is the Pedersen Springbrook where it leaves the Refuge 200 m downstream from the convergences of the spring tributaries (Fig. 1). The Pedersen Springbrook is also distinguished by the absence of western mosquitofish and shortfin molly; a small barrier prevents nonnative fishes access to the Refuge reach of the springbrook.

Purchased in 2001, hundreds of fan palms were removed from the Apcar system in 2007 and the springbrook rerouted to what was judged to be its historic course in 2009. Moapa dace began colonizing the newly excavated 163-m-long springbrook within months after its construction, but density was low at the time of our study and probably below carrying capacity. Streamflow in the Apcar Springbrook had the greatest potential for fluctuation in discharge due to water diversion for municipal use.

The Plummer Springbrook was used in the development and testing of our habitat models because it harbored the greatest density of Moapa dace during our five years of study (unpublished survey data, U.S. Fish and Wildlife Service, Las Vegas Field Office). The Plummer Springbrook has three tributaries converging 45 m upstream from where the springbrook leaves the Refuge at Warm Springs Road. With the assistance of The Nature Conservancy this property and spring province was acquired by the U.S. Fish and Wildlife Service in the late 1990s and a major rehabilitation of the spring province and springbrook occurred in 2006 and 2007. The rehabilitated springbrook is composed of small pools, riffles, glides, and small falls; it also has a public viewing chamber and is the focus of the Refuge’s visitor center.

### Hydrodynamic Modeling

We simulated the hydraulic conditions in the three Refuge springbrooks with River2D [22], a two-dimensional (2D), depth-averaged model [23]. Developed for streams and rivers, River2D has been extensively verified [24]–[26]. One of River2D’s strengths is its variable-size mesh that can be optimized to obtain fine-scale details in areas of interest. Given the small size of the Refuge springbrooks, we constructed a mesh with 8–12 cm resolution to accurately discern hydraulic features associated with Moapa dace. We avoided one- and three-dimensional models because they produce data too coarse- (1-D) or fine-scale (3-D) to efficiently model Moapa dace foraging habitat (i.e., <1 m^2^), while providing the flexibility to map and compare habitat across the entire Refuge [27]. Three products output by River2D are depth-averaged velocity, water depth, and Froude number, calculated at each intersection (node) of a triangulated irregular mesh, for a given flow. The Froude number is a dimensionless hydraulic variable that can objectively identify pool, riffle, and glide features [28], [29].

To insure confidence in the predictability of our 2-D-hydrodynamic model, we followed the methodology and steps in the on-line manual http://www.river2d.ualberta.ca and real-life applications [30], [31]. Refer to File S1 for details related to bathymetry, substrate, or water temperature; File S2 for hydrodynamic boundary conditions; and File S3 for a calibration chart of Plummer Creek (0.071 cms). We could not verify simulations that were higher or lower than the baseflows for each springbrook since their flows were unwavering during and proceeding the study period. Nor could we manually change the inflows at each springhead for verification purposes due to the endangered status of Moapa dace. Thus, we relied exclusively on the calibration of the baseflow simulations and the depth-averaged St.Venant equations [22] to reach equilibrium (inflow equals outflow) for each flow simulation (see Hydrodynamic and Habitat Modeling Accuracies section in Discussion for details as to how this may affect our simulations).

### Snorkel Surveys

Three snorkel surveys were conducted during the spring of 2009 on Plummer Springbrook between April 20 and May 28. Spaced approximately two weeks apart, snorkel surveys covered the entire Plummer Springbrook from the spring head to the culvert, located at the Refuge boundary (Fig. 1). Snorkel surveys began at the downstream of the springbrook as it left the Refuge and the snorkeler crawled upstream until a subject Moapa dace was sighted. After it was judged the fish was unaffected by the snorkeler’s presence, its location was marked on a map as accurately as possible. Fish habitat use is influenced by size and life stage [32] and for our model we used dace ranging from about 40 to 85 mm fork length (FL), the largest observed on the Plummer Springbrook. Fish 40 mm FL were in the late juvenile stage [12], but used the same habitat as adults. For model construction, we drew polygons around dace locations to create occupied patch boundaries, with larger dace clusters producing the biggest patch boundaries. All locations outside of occupied patch boundaries were considered empty since no dace were observed in the snorkel surveys. A map of Moapa dace habitat was completed by joining the presence-absence polygons into one continuous surface representing Plummer Springbrook from the spring head to the Refuge boundary, with no areas unsurveyed.

Three follow-up snorkel surveys were conducted in the next 18 months: January 30, 2010; August 10, 2010; and January 30, 2011. The last survey date was unique because all three Refuge springbrooks were surveyed, while only Plummer Springbrook was surveyed on the other two dates. Thus, the first two snorkel surveys were used to calibrate and verify the habitat model in Plummer Springbrook, while the third survey was used to verify the model on Pedersen and Apcar springbrooks following extrapolation of the model. This approach allowed us to perform an independent verification of the habitat model over both space and time.

### Environmental Database

We constructed an environmental database for habitat modeling by georeferencing all data to a common coordinate system (UTM, Zone 11, NAD83), with each variable rendered as a grid with 12X12-cm (0.014 m^2^) resolution (Table 1). Five predictor variables were created from River2D and field surveys for each springbrook; the principal variables were water depth (DEP), velocity (VEL), Froude number (FRD), substrate (SUB3), and water temperature (TMP). Additional variables were created for modeling purposes through the aggregation of substrate and Froude values into different size classes. Specifically, Froude number was reclassified into pool, riffle, and glide classes with FRD thresholds (pool: Fr <0.18; riffle >0.41; with glide intermediate) [29], while six substrate classes (fines, small gravel, medium gravel, large gravel, cobble, boulder) were aggregated into three classes (fines, gravels, cobble/boulder). Lastly, higher-order terms (e.g., quadratic, cubed) were created for each continuous variable for curvilinear model testing.

**Table 1:** Predictor variables used for Moapa dace habitat modeling.

Variable	Type	Description
VEL	Continuous	Depth-averaged velocity (m/s) obtained from 2D hydrodynamic model
DEP	Continuous	Water depth (m) obtained from 2D boundary conditions
FRD	Continuous	Froude values greater than 1 are super-critical flow; values <1 are sub-critical flow
SUB3	Categorical	Three substrate classes: 1 = fines, 2 = gravels, 3 = cobbles/boulders
SUB7	Categorical	Seven substrate classes: the three groups (SUB3) are further subdivided by size
TMP	Continuous	Temperature °C

### Habitat Modeling

We used cell-based (raster) modeling [33] and logistic regression [34] to build and test numerous Moapa dace habitat models for Plummer Springbrook. We employed logistic regression because it is well suited for the examination of the relationship between a binary response (i.e., presence or absence) and various explanatory variables [34], [35]. We constructed a set of candidate habitat models for comparison and hypothesis testing with presence/absence snorkel data (spring 2009), physical variables (2D hydrodynamic data and substrate maps), logistic regression, and cell-based modeling. We used ArcGIS (version 9x; Redlands, CA) for database construction, SPSS (Chicago, Ill) for logistic regression, and ARC/INFO GRID (ESRI, 1992) for cell-based modeling.

**Figure 2:**
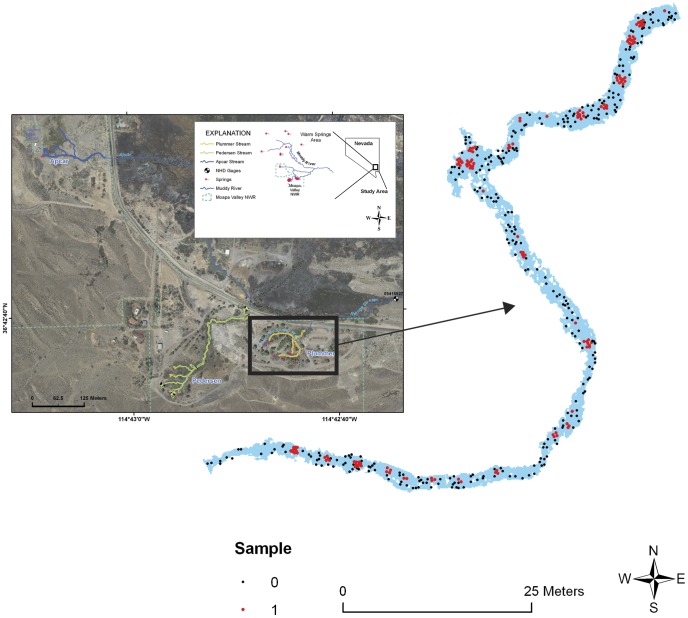
Random sample locations used for model development inside and outside of occupied dace patches in Plummer Springbrook. Snorkel surveys in the spring of 2009 were conducted to determine the locations of Moapa dace (shown in red), while absence locations were generated randomly outside of known dace sites with a GIS (309 absences and 141 presences).

A couple of challenges we faced when developing a model were spatial errors in the observations (∼ 0.5–1 m) and an uneven distribution of dace, reflecting habitat preferences at certain locations. We dealt with spatial errors by randomly generating locations inside of occupied patch boundaries, reasoning that the fish were moving and feeding at the time of observation. We preserved the unequal distribution of dace by generating the same number of random points in each patch as the mean number observed in the snorkel surveys. Lastly, we characterized the larger, unused (background) portion of Plummer Springbrook by generating more absences than presences [36], [37], with a minimum spacing of 12 cm (309 absences versus 141 presences; Fig. 2). Our approach reduced spatial autocorrelation by ensuring that no cell was sampled twice and that its neighboring cells were empty, while capturing habitat preferences through the unequal allocation of random points that were informed by snorkel abundance data. Following the compilation of random points, we attributed each location with its respective environmental features (e.g., velocity, depth) with a GIS.

We evaluated the predictive capability of combinations of covariates on dace occurrence with multivariate logistic regression. Given the field work that had been conducted to date on the Refuge, we held an a priori assumption that a combination of geomorphic features and hydraulic conditions was important for Moapa dace (Table 1). We used backward elimination and the likelihood-ratio test to identify significant covariates, starting with a full model and then progressively removing one or more variables and examining the change in Akaike’s information criterion (AIC) [38]. We checked for nonlinearity between the logit and a continuous variable with quadratic, cubic, and log terms [39]. We evaluated 11 candidate models, comparing their performance with AIC model weights [38], Nagelkerke’s pseudo-R^2^ [40], Hosmer–Lemeshow goodness-of-fit statistic Ĉ [34], a binary classification table [41], and a Receiver Operating Characteristic (ROC) area-under-the-curve (AUC) [42].

### Model Application and Verification

We generated spatially explicit maps of predicted Moapa dace habitat in Plummer Springbrook with cell-based modeling techniques [33], populating each model with its respective predictor variables (grids). We examined model accuracy with snorkel data described previously. We focused only on presence locations for verification purposes since the differences in Moapa dace numbers (∼4X, this paper) on the three Refuge springbrooks were large, reflecting the recent history of habitat modifications and enhancement on each stream, versus the quality of habitat, making a comparison of model commission meaningless among streams.

We constructed a binary habitat map for each model by applying a probability cutpoint (threshold) of 0.3, which we obtained through trial and error during the model development and testing phase on Plummer Springbrook, balancing omission and commission errors [37]. Specifically, grid cells with a probability >0.3 were assigned a value of 1 (habitat), while cells with probabilities ≤0.3 were converted to zero (non-habitat). We used a GIS to overlay dace locations and habitat maps, calculating accuracy as the percentage of dace locations that fell within predicted suitable areas. Since there was some error in assigning locations of dace observed in the field to a map, we considered any dace that fell within two cells (∼24 cm) of an occupied patch to be a correct classification.

We assessed model fit by examining the density of presence locations found within discrete probability classes [31], [37]. Specifically, we created 20% interval classes from the continuous probabilities output from the habitat model, overlaid dace locations, and calculated the density of dace within each probability class (number of dace/cell/probability class). A good fit to the model should be demonstrated by an increasing number of dace locations inside of higher probability classes.

Extrapolating the models to Apcar and Pedersen springbrooks required that we not change the model coefficients or probability threshold that were obtained on Plummer Springbrook, only the predictor grids (substrate, velocity, depth, Froude number). Applying the Plummer Springbrook habitat model to Plummer, Apcar, and Pedersen springbrooks ensured a true test of our habitat model in a spatial and temporal perspective.

### Hydraulic Habitat Simulations

We conducted habitat simulations over a range of flows by ramping up or drawing down the flow in each Refuge springbrook by 30% relative to its baseflow, in 10% increments, calculating the amount of habitat at each flow with the habitat model. We tabulated the amount of predicted dace habitat for each flow simulation and displayed the results in bar graphs. Due to different reach lengths and base flows of the three springbrooks, we standardized our results for comparison purposes in two ways. First, we divided the amount of predicted habitat for each habitat-flow simulation by the length of springbrook, resulting in the amount of predicted habitat per-linear-meter of channel. Second, we divided the difference between each habitat-flow simulation from its base-flow habitat estimate, producing the magnitude of change relative to its baseflow.

## Results

### Hydrodynamic Modeling

Two-dimensional hydraulic simulations for Plummer, Pedersen, and Apcar springbrooks achieved velocity and depth accuracies from 74%–91% (RMSE) and 84%–92%, respectively (see File S3). Each 2D springbrook simulation produced distinct patterns in depths and velocities, with pools and riffles easily discerned by their shapes and profiles (see File S4). Applying Froude thresholds to velocity and depth data revealed that Plummer Springbrook was comprised of 70% pools, 18% glides, and 12% riffles (baseflow = 0.071 cms). In contrast, Pedersen Springbrook was comprised of 50% pools, 33% glides, and 17% riffles (baseflow = 0.108 cms). Lastly, Apcar was comprised of 67% pools, 15% glides, and 18% riffles (baseflow = 0.066 cms).

### Snorkel Surveys

Snorkel surveys in Plummer Springbrook (20 April through 28 May, 2009) revealed that dace were located at similar locations in different surveys, but moved significantly between sites (CV ∼ 60%). However, overall abundance changed little between surveys (<5%), with an average of 141 dace, or 1.1 fish per-linear-meter of stream channel inside the Refuge. The two follow-up snorkel surveys in Plummer Springbrook detected 127 dace on 30 Jan 2010 (0.96 fish/m) and 161 dace on 30 Jan 2011 (1.2 fish/m). In contrast, only 62 dace were detected on Pedersen Springbrook on 30 Jan 2011 (0.26 fish/m) and 34 dace on Apcar Springbrook (0.21 fish/m). Thus, Plummer Springbrook had ∼4 times the number of dace per-linear-meter of springbrook than the other two refuge streams.

### Habitat Modeling

**Figure 3:**
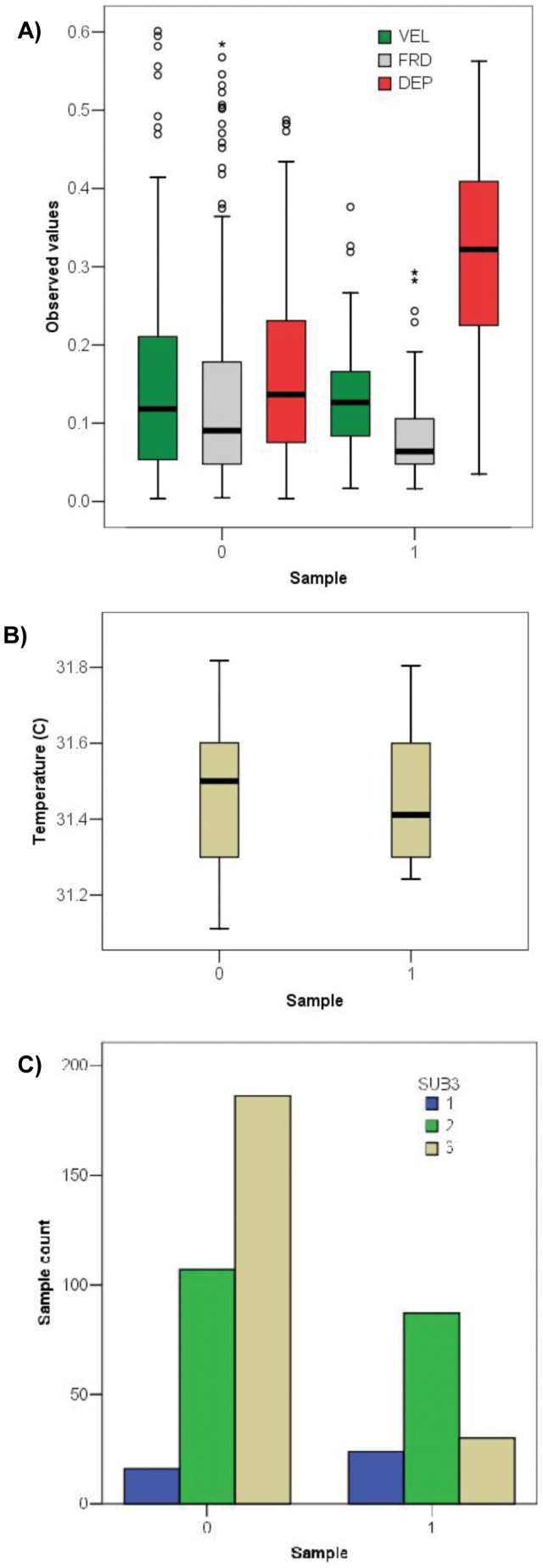
Box-and-whisker plots and bar graphs display the range of environmental values found at 450 sample locations in Plummer Springbrook (see Fig. 2). Panel A displays the distribution of velocity (m/sec), depth (m), and Froude values; panel B shows temperature values; panel C portrays the number of presence or absence sample locations found within three substrate classes (1 = fines, 2 = gravels, 3 = cobble/boulder).

We saw distinct differences in velocity and depth conditions selected by Moapa dace, as compared to random background locations, in Plummer Springbrook at a baseflow of 0.071 cms (Fig. 3A), and a small difference in temperature (Fig. 3B). The further apart each group’s medians, the stronger the evidence for habitat selectivity, while the closer the quartiles are within a group (i.e., 0 or 1), the smaller (more specific) the niche. The largest differences in median values between each sample group, listed in descending order of importance, were water depth, Froude number, stream temperature, and velocity. For the categorical variable substrate (Fig. 3C), the largest number of absence locations occurred inside cobble/boulder areas, while the largest number of presence locations occurred inside gravel areas.

Univariate logistic regression revealed that water depth had the closest association with dace locations during the spring of 2009 (Table 2; baseflow = 0.071 cms), followed in descending order of importance by substrate (3 classes), Froude number (continuous), velocity, and water temperature. Water depth obtained a good fit across 10 probability deciles (Ĉ = 0.5), explained 37% of the variability, achieved 75.1% overall classification accuracy (binary; probability threshold = 0.3), and achieved an AUC of 0.82. The next closest univariate was substrate, with an AUC of 0.71. Of the univariables, only temperature had a non-significant AUC.

**Table 2:** Model results for univariate and multivariate logistic regression, listed from best to worst according to AIC score (*n* = 450; 309 absences and 141 presences).

Model	LL	NPar	AIC	ΔAIC	w	Ĉ	R^2^	OA	AUC	Variables
1	407.461	8	423.461	0.000	0.484	0.685	0.403	76.20	0.838	*DEP, FRD, SUB3
2	409.241	8	425.241	1.780	0.199	0.608	0.399	76.20	0.835	*DEP, VEL, SUB3
3	421.035	3	427.035	3.574	0.081	0.499	0.372	75.70	0.826	*DEP, FRD
4	419.037	4	427.037	3.576	0.081	0.797	0.377	75.05	0.826	*DEP, VEL, FRD
5	407.284	10	427.284	3.828	0.072	0.821	0.403	76.30	0.838	*DEP,FRD,TMP,SUB3
6	421.916	3	427.916	4.455	0.052	0.499	0.370	75.10	0.823	DEP
7	408.963	10	428.963	5.502	0.031	0.488	0.400	76.55	0.835	*DEP,VEL,TMP,SUB3
8	494.787	4	502.787	79.326	0.000	NA	0.188	69.45	0.708	SUB3
9	491.44	8	507.440	83.979	0.000	NA	0.197	68.65	0.724	SUB7
10	519.993	2	523.993	100.532	0.000	0.040	0.118	59.50	0.664	FRD
11	522.173	3	528.173	104.712	0.000	0.247	0.112	61.65	0.670	VEL
12	527.814	4	535.814	112.353	0.000	NA	0.096	59.60	0.598	FRD3
13	559.568	1	561.568	138.107	0.000	0.000	0.000	54.90	0.500	TMP

Statistics presented are twice the negative log-likelihood value (−2L), the number of parameters (NPar), change in AIC score when compared to the best model (ΔAIC), AIC model weight (w), Hosmer-Lemeshow goodness-of-fit statistic (Ĉ), Nagelkerke pseudo R-squared (R^2^), overall classification accuracy (OA), ROC area-under-the-curve (AUC), and the principal variables in each model (higher-order terms not shown. For variable descriptions, see Table 1; * denotes the variable that had the greatest influence on the model’s log likelihood. Quadratic terms are not shown in the Variables field.

Of the 13 models we tested (Table 2) the top performer (according to AIC) contained a depth and substrate variable, plus a Froude variable (Model 1). Model 2 was also strongly supported by AIC (ΔAIC = 1.78), but contained a velocity variable in place of the Froude variable. We could not pair velocity into most models that contained Froude due to high colinearity, but we could pair depth with Froude - even though Froude incorporates depth into its computation. There was moderate support for Models 3–7 (ΔAIC between 3 and 6), with no support for the remaining six models (ΔAIC >79). The critical variable that resulted in the large gap in AIC scores between Models 1 thru 7 and Models 8 thru 13 was depth. Whenever depth was in a model, it was either strongly (Models 1,2) or moderately (Models 3–7) supported. No other covariate influenced the multivariate models to the magnitude of depth, with substrate a distant second, followed by Froude number, velocity, and temperature. Depth was also the best univariate model (Model 6), achieving equal model-fit statistics as the top five models, with the exception of its AIC score (ΔAIC = 4.455). The two temperature models (5 and 7) were only moderately supported by AIC, but Model 5 achieved the best overall model fit (Ĉ = 0.821) and tied model 1 for best R^2^ (0.403) and AUC (0.838), while Model 7 obtained the best overall classification accuracy (76.6%), indicating temperature played a small role in dace habitat selection in Plummer Springbrook.

We selected Model 2 for model extrapolation into Apcar and Pedersen springbrooks, and for hydraulic-habitat simulations (i.e., ramping up and drawing down flows), because it was strongly supported by AIC, achieved a reasonably good model fit (Ĉ = 0.608), and velocity is easier to interpret than the Froude number. We also found little difference in performance between these two models from an accuracy or spatially explicit perspective (model parameters for Models 1 and 2 are listed in Table 3). We retained covariates in Models 1 or 2 if they improved the overall fit of the model (Ĉ), regardless of statistical significance (Table 3). While quadratic terms improved the fit of both models, indicating non-linear relationships, logarithmic and cubic functions failed to improve model fit.

**Table 3:** Model parameters and coefficients for Model 1 (top) and Model 2 (bottom): outputs were obtained from multiple logistic regression on Plummer Creek, with samples collected in the spring of 2009 (*n* = 450; 309 absences and 141 presences).

Model 1					
Variable	*B*	S.E.	Wald	df	Sig.
DEP	12.745	4.402	8.383	1	0.004
DEP_2	−8.956	7.822	1.311	1	0.252
FRD	4.778	6.065	0.621	1	0.431
FRD_2	−22.941	20.594	1.241	1	0.265
SUB3 (reference)			10.44	2	0.005
SUB3 (class 1)	1.134	0.442	6.595	1	0.01
SUB3 (class 2)	0.8	0.282	8.031	1	0.005
Constant	−3.838	0.626	37.634	1	0
**Model 2**					
DEP	13.935	4.426	9.913	1	0.002
DEP_2	−10.923	7.746	1.989	1	0.158
SUB3 (reference)			10.272	2	0.006
SUB (class 1)	1.126	0.447	6.345	1	0.012
SUB (class 2)	0.796	0.282	7.979	1	0.005
vel252b	4.238	5.135	0.681	1	0.409
vel252b_2	−15.174	14.272	1.13	1	0.288
Constant	−4.047	0.604	44.914	1	0

See Table 1 for variable definitions; variables with an underscore (e.g., Dep_2) are squared terms.

Interpretation of the odds ratios (exp β) and model coefficients for Models 1 or 2 provided information about the habitat preferences of dace. Specifically, dace were approximately three times as likely to occur on sandy substrates as a cobble-boulder substrate, and approximately two times as likely on a gravel surface. Interpretation of the squared terms revealed that in small springbrooks dace about 40 to 85 mm FL preferred water depths between 0.64 and 0.71 m, a Froude value of 0.1 (non-stagnant pool), and a velocity of 0.14 m/s. These values changed slightly when other models were examined, but they were not as well supported by AIC as Models 1 or 2.

### Model Application and Verification

Model 2 produced a mean probability for dace habitat in Plummer Springbrook of 0.21 (baseflow 0.071 cms), with a maximum of 0.83 and a minimum of 0. Applying a habitat probability threshold of 0.3, 26.8% (0.007 ha) of Plummer Springbrook was predicted to be dace habitat at a baseflow of 0.069 cms (see File S4). Model 2 achieved 88% accuracy in August 2010 when challenged with independent snorkel data (22 out of 25 sites correct), 90.5% accuracy in January 2010 (19 of 21), and 91.1% in January 2011 (41 out of 45).

**Figure 4:**
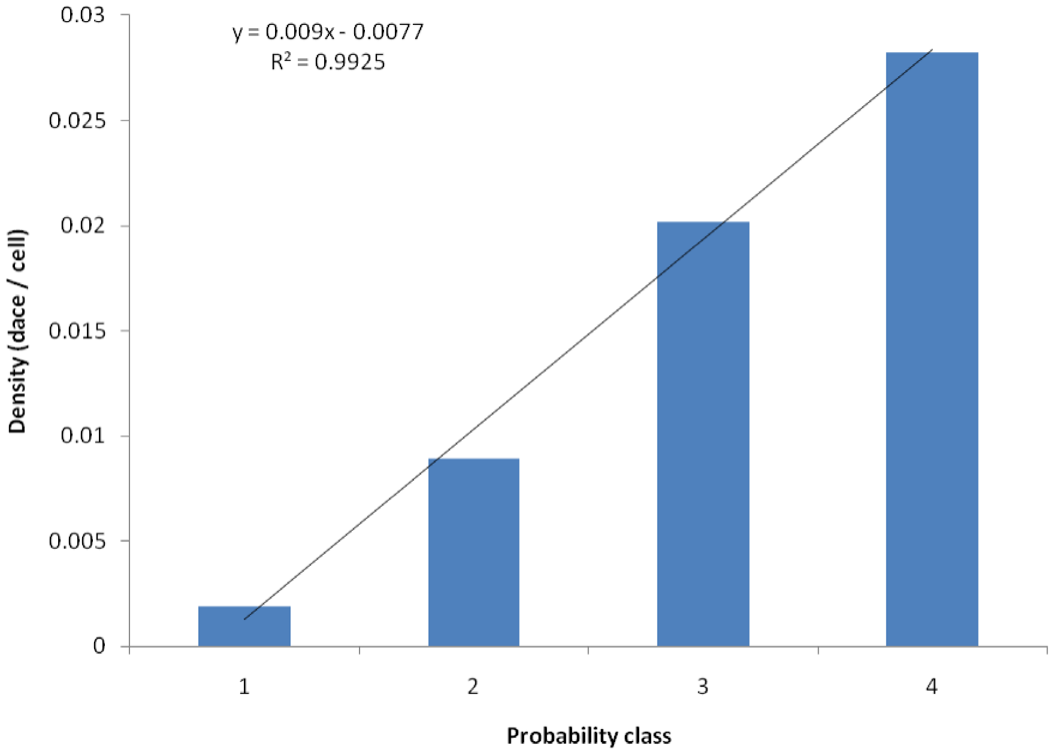
The relationship between Moapa dace density and four probability classes in Plummer Creek, as output by Model 2. Probability classes are 1 (0–20%), 2 (20.1–40%, 3 (40.1–60%), and 4 (>60%). Dace densities were obtained by averaging three back-to-back snorkel surveys (spring of 2009), counting the number of dace within each probability class, and dividing by the number of cells (0.0144 m^2^) found within each probability class.

Model 2 obtained a good fit when we examined dace density per probability class in Plummer Springbrook, using snorkel data in the spring of 2009 (Fig. 4). For this analysis we merged probability classes 4 and 5 since the model’s probabilities topped out at 84%, producing too few cells or fish observations in class 5 to stand alone. Thus, we calculated dace densities inside four probability classes: 0–20%, 20.1–40%, 40.1–60%, and >60%. The following equation describes the relationship between dace density and the four probability classes in Plummer Springbrook.
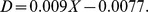
where *D* is the density of dace per cell (0.0144 m^2^) for a given probability class. Our density estimate for Plummer Springbrook appeared to represent future dace conditions too since the numbers of dace in the two future snorkel surveys bracketed the numbers observed in the spring of 2009, with the locations approximately the same.

The mean probability of dace occurrence in Pedersen Springbrook, using Model 2, was 22.2% (baseflow 0.108 cms), with a maximum probability of 85.3%, and a minimum of 0%. Applying a 30% probability threshold resulted in 29.3% (0.013 ha) of the springbrook predicted to be dace habitat (see File S4). When we challenged the habitat model to independent snorkel data collected in January 2011, the model achieved 84.6% accuracy (22 of 26 sites correctly classified). The mean model probability for Apcar Springbrook, using Model 2, was 30.8% (baseflow 0.066 cms), with a maximum probability of 86% and a minimum of 0%. Applying a 30% probability threshold resulted in 42.7% (0.013 ha) of Apcar Springbrook predicted to be dace habitat (see File S4). When we challenged the habitat model to independent snorkel data collected in January 2011, the model achieved 90% accuracy (18 of 20 sites).

### Hydraulic Habitat Simulations

**Figure 5:**
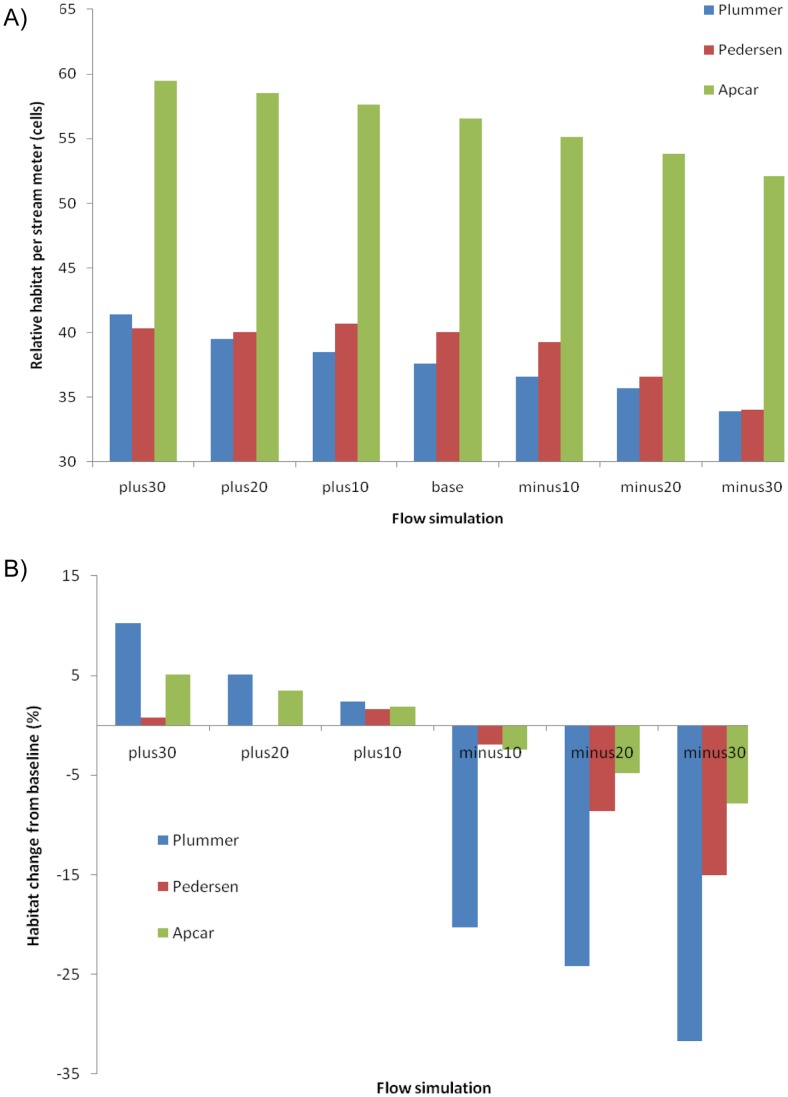
Habitat-discharge relations among Plummer, Pedersen, and Apcar creeks, in 10% flow increments. In panel A, the amount of predicted habitat by flow is presented after standardizing the data by stream length. In panel B, the relative change in habitat in relation to baseflow was calculated in 10% flow increments.

When we supplied the habitat model with seven flows, starting at a 30% increase over baseflow and then descending in 10% increments - until a 30% reduction was achieved - habitat (per-linear-meter of stream channel) appeared to decrease steadily in Plummer and Apcar springbrooks (Fig. 5A). This pattern was not the same for Pedersen Springbrook, where the maximum habitat was obtained at a 10% increase over baseflow, before leveling out. The amount of predicted habitat per-linear-meter of springbrook revealed that Apcar Springbrook is expected to produce the most dace habitat over the range of flows. The slope of the increase for Plummer Springbrook appeared similar to Apcar, but the amount of predicted habitat per-linear-meter of channel was approximately 30% less. In contrast, Plummer and Pedersen springbrooks had different slopes (reactions), but the amount of predicted habitat per-linear-meter of springbrook was similar at the top and bottom of the flow simulations. However, Pedersen Springbrook appeared more responsive to flows between minus 20% and plus 20% compared with Plummer Springbrook. When we simulated how dace habitat in each springbrook would change in relation to its baseflow prediction (Fig. 5B), Plummer Springbrook appeared the most sensitive, with potential losses of approximately 30% and increases of 10%. Pedersen Springbrook appeared to be the second most sensitive to flow modifications, with potential habitat losses of 15% and gains of 2%. In contrast, Apcar Springbrook gained or lost approximately 5% of its predicted dace habitat in relation to its baseflow, indicating it was least sensitive to flow alteration.

## Discussion

### Hydrodynamic and Habitat Modeling Accuracies

The accuracy rate of our 2D hydrodynamic flow simulations ranged from 73–91%, under baseflow conditions, which is consistent with other 2D studies on large and small streams [26], [30], [43]. We were unable to calibrate or validate non-baseflow simulations given the unvarying springheads over the study period. Calibration typically involves changing mesh configuration or roughness values to achieve closer agreement between simulated and measured water surface elevations and velocities [23], [30]. Thus, our flow simulations may have bias that could affect habitat classification, but the baseflow had good verification results and it was the midrange of our flow simulations. To our knowledge these are some of the smallest streams where 2D fish-habitat modeling has been conducted and we are satisfied given the 85–91% accuracies Model 2 achieved with temporally and spatially independent snorkel-survey data. Furthermore, the excellent linear fit between the model’s probability classes and dace densities demonstrated that the model provided useful information about the quality of dace habitat (i.e., higher dace numbers informed the model of preferred hydrogeomorphic conditions).

### Habitat-flow Simulations

Plummer and Apcar springbrooks produced proportionately more habitat as flows increased, while Pedersen springbrook reached a plateau after a 10% increase, suggesting a geomorphic constraint. In contrast, Plummer and Apcar springbrooks appeared relatively unconstrained by geomorphology and thus dace might benefit from increased flows. Conversely, habitat simulations consistently showed in each springbrook that reduced flows produce less Moapa dace habitat. A reduction in habitat is typically followed by a reduction in population number, thus the information in this study is important when considering population dynamics in relation to streamflow [44].

Because Refuge springbrooks are close to spring heads, Refuge habitat experienced a very narrow temperature range and our analysis garnered only moderate support for the two temperature models (Table 2, Models 5,7). Had we had the opportunity to study Moapa dace in its historic range in the Muddy River, where waters are cooler, the influence of temperature in our models would likely be greater because larger, older fishes frequently inhabit cooler water [45], [46]; a phenomena previously observed in Moapa dace [12]. A reduction in springflows on the Refuge or Muddy River could result in stream cooling [47], which may reduce the area currently suitable for rearing, foraging, and spawning (26°−32°C).

### Detection

Moapa dace have patchy distribution and congregate in predictable hydraulic conditions, as defined by our model. Foraging primarily upon drift [12], Moapa dace presumably select hydraulic conditions that promote optimal foraging [48], hence their patchy distribution. They are also quite transient, frequently moving among patches [14], with an average movement of 68 m between bi-monthly sampling events, and ∼30% leaving the refuge entirely (Mark Hereford, USGS Biologist, personal communication). As more information is gathered through tagging and genetic analysis, we will gain a better understanding of dace migration rates on and off the refuge, particularly at finer temporal and spatial scales. Until this occurs, we chose not to incorporate detection probabilities into our modeling approach [49].

Habitat selection can be density dependent with only higher quality habitat used when population numbers are low [50], [51]. The Plummer Springbrook was inhabited by well over 50% of the Moapa dace population during the period of our study and presumably virtually all available habitats were occupied during our snorkel surveys. We are confident based upon our extrapolation tests (temporally and spatially) that the habitat model we developed for Plummer Springbrook, and extrapolated to Pedersen and Apcar springbrooks, captured the essential features that comprise dace habitat. Namely, water depth, substrate composition, Froude number, and velocity, with temperature a distant last.

### Habitat Restoration-Rehabilitation

Habitat rehabilitation in the three Refuge springbrooks was crudely modeled on sites observed to support congregations of foraging Moapa dace before they became restricted to the Refuge (Unpublished report: G. Gary Scoppettone). Most sites were in the upper Muddy River where the catchment basin intermittently floods, producing flows well beyond the historic 1.1 m^3^/s attributed solely to thermal springs [9]. The cut and fill alluviation produced by intermittent flooding most likely built and destroyed Moapa dace habitat in the main-stem Muddy River in a dynamic process that has occurred for thousands of years. These dynamic flooding-erosion processes generally decrease in an upstream direction [52], thus catchments with smaller or reduced drainage areas are not as dynamic. The Refuge springbrooks have all been cut off from their respective sub- catchment basins and thus the quality of Moapa dace habitat will likely degrade in time due to emergent and submergent vegetation. Without intermittent flooding to maintain or generate new dace habitat, the Refuge springbrooks will need to be continually monitored for habitat quality, with habitat restoration conducted on an as-needed basis.

Our habitat models provide targets and thresholds for managers in the development, evaluation, and monitoring of dace habitat. For example, the amount of predicted habitat from our models can be used as an indicator of the effectiveness of a restoration or enhancement activity. In addition, changes in habitat quantity or quality could be assessed by calculating habitat prior to and after a restoration or enhancement activity, calculating the mean probability for a given reach, or habitat quantity through application of a probability threshold (30% for our models). It is also possible to use the habitat models to simulate the benefits of a given restoration or enhancement activity before committing the funds for on-the-ground efforts to implement the proposal. Simulating an enhancement activity would involve modifying the bathymetry, rerunning the 2D hydraulic model, and recomputing habitat. One could compare multiple scenarios when determining the most optimum use of resources for the restoration or enhancement of dace habitat. The final evaluation criterion for any project should be the number of dace observed prior to and following a restoration or enhancement activity, with the models providing guidance on the achievement and monitoring of dace habitat over space and time.

### Conclusion

This study indicates that a reduction in spring discharge within Moapa Valley National Wildlife Refuge will cause a reduction in important refugial habitat for Moapa dace, and may exacerbate native-nonnative interactions [53], [54]. The Muddy River’s carbonate aquifer is being closely monitored to prevent breaching its sustainability (personal communication, Lee Simons, U.S. Fish and Wildlife Service, Las Vegas, Nevada). However, there are concerns that pumping from the aquifer may cause an unintended overdraft and a reduction in spring discharge [21]. Another looming threat to sustaining the Muddy River’s carbonate aquifer is global climate change. The southwest is expected to get warmer and drier in the next century, with spring and summer streamflows predicted to be significantly reduced [5], [6]. While it is unknown how climate change will affect the groundwater in the vicinity of the Refuge, it will probably decrease as the climate warms and dries. Our model provides important information to managers charged with protecting and recovery of Moapa dace in an era of potential reduction in thermal spring discharge feeding the Muddy River.

The focus of this study was Moapa dace, but our results have implications for seven other aquatic species listed as sensitive in the Muddy River ecosystem [11]. Each species has its own specific habitat requirements, by life stage, but they all share the Muddy River ecosystem and a threat to one species is a concern for all. We have shown that reduced flows on the Refuge will threaten Moapa dace habitat, while increased flows would provide benefits. Until we know more about the habitat preferences of all aquatic species in the Muddy River ecosystem, a water conservation strategy that minimizes any net loss in habitat is desirable.

## Supporting Information

File S1 **Background information on substrate and bathymetric surveys.**
(DOC)

File S2 **Detailed boundary conditions for River2D hydraulic simulations.**
(DOC)

File S3 **2D hydrodynamic model calibration and verification charts for each springbrook.**
(DOC)

File S4 **2D hydrodynamic model output and habitat maps for each springbrook.**
(DOC)

Video S1 **A typical sandy-bottom plunge-pool habitat selected by Moapa dace.** Identified by their fusiform body and deeply forked tail with black spot at its base, Moapa dace are actively working the water column for drift items. Also in the video are Moapa White River springfish identified by their square tail. Both species are thermal endemic, typically occurring in water temperatures from 26 to 32°C and are restricted to the headwaters of the Muddy River, Clark County, Nevada where the river originates from a series of thermal springs. Video provided by Pete Rissler (U.S. Geological Survey).
(MP4)
