# Supplementary material for: An Ecohydraulic Model to Identify and Monitor Moapa Dace Habitat
Source: PLoS One. 2013 Feb 7;8(2):e55551. doi: 10.1371/journal.pone.0055551 (PMC3567127; doi:10.1371/journal.pone.0055551)
Supplement: File S1 — Background information on substrate and bathymetric surveys. (DOC) [file pone.0055551.s001.doc]

**S1. Substrate, bathymetric, and water-temperature surveys**

Substrate Mapping

There are many methods available to determine substrate classes in streams. The use of Wolman pebble counts [S1.1] and video imaging [S1.2] are two widely used examples. We decided to incorporate multiple classification methods into our survey to complete substrate classification in these small streams. We used video and still imagery combined with the use of RTK GPS and reference frames to complete our assessment of substrate. Following the creation of all seven model runs for each of the three complexes, substrate video data was collected and polygons created for each area to be included in the resulting habitat models. The video substrate dataset creation started with the collection of Hi Definition (HD) video with an Olympus Stylus 6020 waterproof camera. A reference frame is placed on the bottom and a RTK point is collected in the middle of the frame. The video is recorded to cover the extent of the reference frame every 1 meter, or 50 cm in Apcar, and ~2 meters -depending on the stream width - both Plummer and Pedersen creeks. This technique is repeated moving up the stream with both still photographs and RTK points collected on large edge features - such as boulder fields. We collected 168 videos and images in Plummer Creek, 202 in Pedersen Creek, and 246 in Apcar Creek.

The resulting video files were played back and still JPEG images were exported to cover an area forming a quadrant around the reference frame. These photos were imported into ImagePro Plus and the images had a reference scale set by measuring either the frame or other items in the image that have a known dimension in the frame [S1.3]. A reference grid was overlain on the image and multiple measurements were made to help determine the dominant substrate class. We used the tools available in ImagePro to draw lines across the substrate that was larger than the reference grid to determine if the larger substrate was the dominant or subdominant class. This process was repeated for all the collected images in each of the three study areas.

Using the measured reference images and videos as a guide, points were created showing the dominant substrate in each quadrant as identified from the videos. The RTK center-frame reference points were attributed with the substrate information and merged with the surrounding point data. Polygons were drawn to enclose the like substrate types and the file was cleaned in ArcMap. Each polygon was attributed with substrate point data and verified in ArcMap.

#### Bathymetric Surveys

#### Our 2D hydraulic models required a continuous, high-resolution digital elevation model (DEM) of the Moapa stream complex. We constructed a DEM for modeling purposes by combining LiDAR and ground-survey data. The Southern Nevada Water Authority conducted two LiDAR surveys in 2007 and 2010, with one-meter point spacing. In addition, the U.S. Geological Survey collected bathymetric and elevation data on the ground with a Real Time Kinematic (RTK) GPS (Trimble 5700) base system, with 9-cm accuracy. Technicians waded streambeds with backpack-mounted antennas, receiving correctional signals from an RTK base station positioned over a benchmark overlooking the Refuge. We established the benchmark by linking to another existing benchmark (WSBM-f) located at the Iverson flume stream gage [S1.4]. This new control point ensured that the correction signal to the RTK rovers covered the entire study area. To ensure maximum accuracy, the benchmark was established by collecting RTK GPS data at a fixed position for eight hours, after which the data were sent to National Oceanic and Atmospheric Administration’s (NOAA) Online Positioning Service (OPUS) to bring the new reference point into alignment with WSBM. We use the RTK GPS data for bathymetric surveys, creation of shoreline polygons, calibration and verification data, and for boundary conditions used by the 2D model. The bathymetric data were converted into Shapefiles, DEMs, and triangulated irregular network (TINs), which were imported into River2D’s bed-file program for modeling purposes. All data were projected into a common reference system (UTM, NAD83).

#### Stream Temperature Surveys

On July 20, 2009, a stream temperature survey was conducted on Plummer Springbrook inside the Refuge. Staff used a calibrated thermometer to collect water temperatures on the surface and bottom of Plummer Springbrook in ~ 3-m intervals, starting at the springhead and ending at the culvert on the Refuge boundary. The water-temperature survey started at 20:45 AM and ended at 12:05 PM, with 48 temperatures collected on the mainstem Plummer Springbrook. Conditions on the Refuge were seasonal (clear and hot), with a springbrook flow of ~2.5 cfs. We observed less than a 1% difference in surface and bottom stream temperatures, indicating a well mixed stream. Additional, follow-up temperature surveys were conducted in 2011 and 2012, but using a temperature filament (DTS) rather than a hand-held thermometer, to determine if the temperatures we observed in the summer of 2009 were representative. Detailed analyses (will be presented in another publication) revealed that the temperatures we recorded in the summer of 2009 were representative in both pattern and magnitude as water temperatures observed in 2011 and 2012. The stability of springbrook temperatures mirrored the almost unwavering spring discharge we observed over the study period, indicating a very stable system from a hydrodynamic and water temperature perspective.

**References**

S1.1. Olsen DS, Roper BB, Kershner JL, Henderson R, Archer E (2005) Sources of variability in conducting pebble counts: their potential influence on the results of stream monitoring programs. J Am Water Resour As 41: 1225-1236.

S1.2. Warrick JA, Cochrane GR, Yael S, Gelfenbaum G (2008). Nearshore Substrate and Morphology Offshore of the Elwha River, Washington. Northwest Sci 82, Special Issue.

S1.3. Media Cybernetics (2001) Image-Pro Plus Reference Guide For Windows. Silver Spring: Media Cybernetics, Inc. 686 p.

S1.4. Beck DA, Ryan R, Veley RJ, Harper DP, Tanko DJ (2006) Water-Surface Elevations, Discharge, and Water-Quality Data for Selected Sites in the Warm Springs Area near Moapa, Nevada. USGS Open-File Report 2006-1311. Available: [http://pubs.usgs.gov/of/2006/1311/](http://pubs.usgs.gov/of/2006/1311/  )  Accessed 2 July 2012.
