# Supplementary material for: An Ecohydraulic Model to Identify and Monitor Moapa Dace Habitat
Source: PLoS One. 2013 Feb 7;8(2):e55551. doi: 10.1371/journal.pone.0055551 (PMC3567127; doi:10.1371/journal.pone.0055551)
Supplement: File S2 — Detailed boundary conditions for River2D hydraulic simulations. (DOC) [file pone.0055551.s002.doc]

**S2. Boundary conditions for River2D hydraulic simulations**

Pedersen Springbrook boundary conditions for 7 hydraulic simulations.

| Model Run | Flow (cfs) | Flow (cms) | Ending WSE (m) | Model Outflow (cms) |
| --- | --- | --- | --- | --- |
| +30 | 3.276 | .093 | 532.750 | .092 |
| +20 | 3.024 | .086 | 532.700 | .085 |
| +10 | 2.772 | .078 | 532.690 | .078 |
| Base flow | 2.520 | .071 | 532.582 | .070 |
| -10 | 2.294 | .065 | 532.680 | .064 |
| -20 | 2.100 | .059 | 532.669 | .058 |
| -30 | 1.760 | .050 | 532.655 | .049 |

| Model Run | Flow (cfs) | Flow (cms) | Ending WSE (m) | Model Outflow (cms) |
| --- | --- | --- | --- | --- |
| +30 | 4.940 | .140 | 541.350 | .140 |
| +20 | 4.560 | .129 | 541.315 | .129 |
| +10 | 4.180 | .118 | 541.297 | .117 |
| Base flow | 3.800 | .108 | 541.258 | .107 |
| -10 | 3.420 | .097 | 541.248 | .095 |
| -20 | 3.020 | .086 | 541.243 | .085 |
| -30 | 2.660 | .076 | 541.239 | .074 |

Plummer Springbrook boundary conditions for 7 hydraulic simulations.

Apcar Springbrook boundary conditions for 7 hydraulic simulations.

| Model Run | Flow (cfs) | Flow (cms) | Ending WSE  (m) | Model Outflow (cms) |
| --- | --- | --- | --- | --- |
| +30 | 3.029 | .086 | 533.765 | .085 |
| +20 | 2.796 | .079 | 533.745 | .079 |
| +10 | 2.563 | .073 | 533.730 | .072 |
| Base flow | 2.330 | .066 | 533.720 | .066 |
| -10 | 2.097 | .059 | 533.710 | .059 |
| -20 | 1.864 | .053 | 533.705 | .052 |
| -30 | 1.631 | .046 | 533.685 | .046 |
