# Supplementary material for: An Ecohydraulic Model to Identify and Monitor Moapa Dace Habitat
Source: PLoS One. 2013 Feb 7;8(2):e55551. doi: 10.1371/journal.pone.0055551 (PMC3567127; doi:10.1371/journal.pone.0055551)
Supplement: File S3 — 2D hydrodynamic model calibration and verification charts for each springbrook. (DOC) [file pone.0055551.s003.doc]

**S3. 2D hydrodynamic model calibration and verification charts**


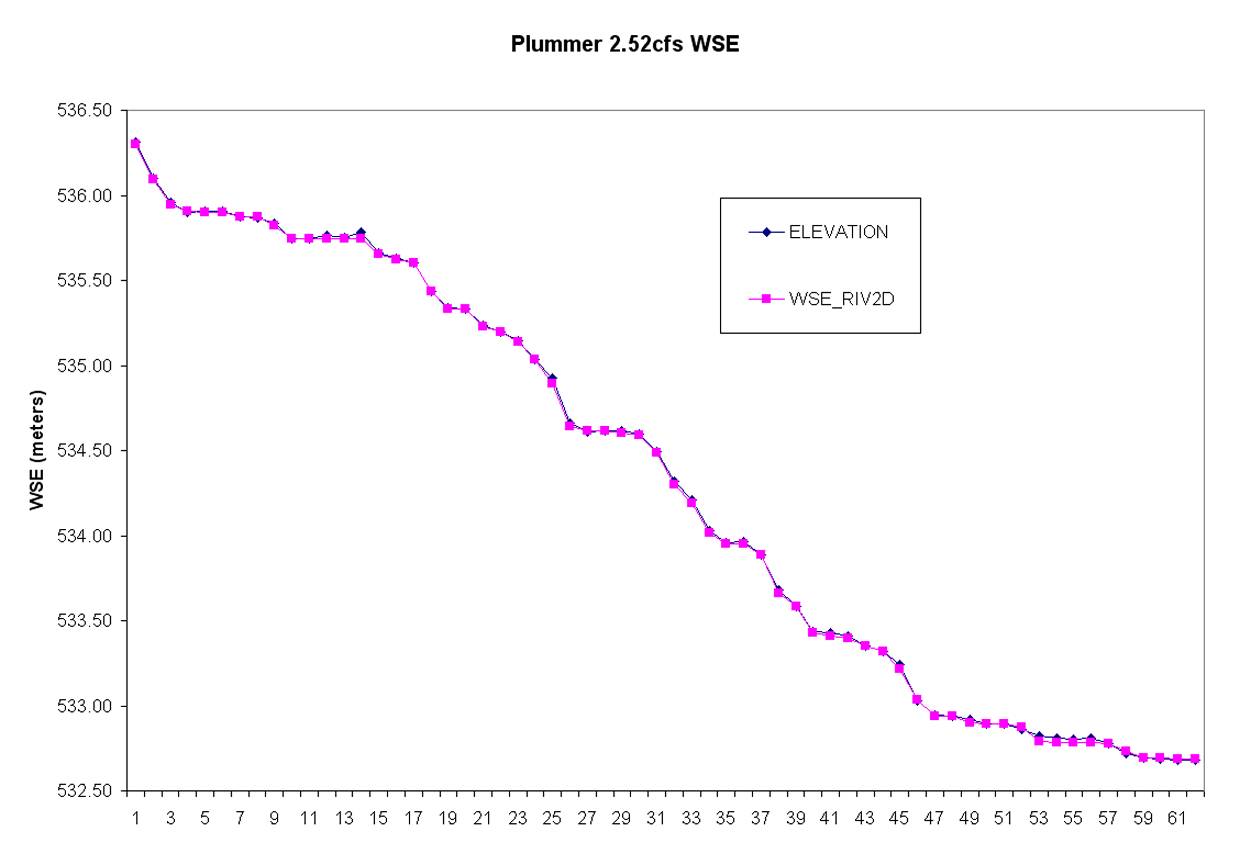


Figure S34.1. A cCalibration chart for Plummer SpringbrookCreek at a 0.071 cms2.52-cfs baseflow. Elevations were collected with a real-time kinetic GPS unit, with centimeter accuracy, overlaid on water-surface elevations output by River2D.


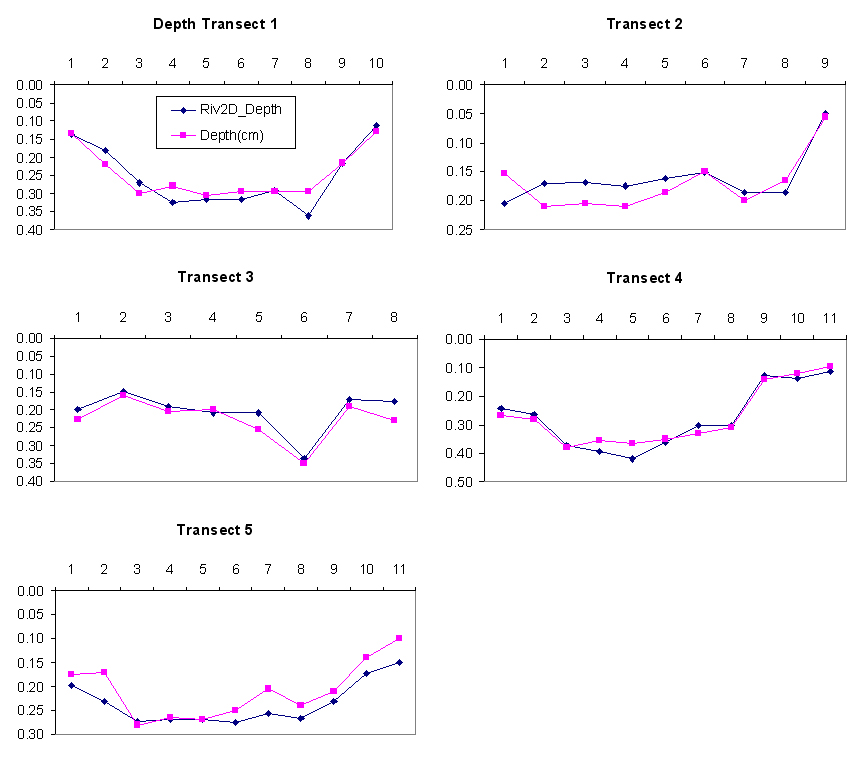


Plummer depth (above line) and velocity (m/s) transects output from the River2D and an acoustic Doppler velocity meter (ADV) for comparison purposes. The root mean square error (RMSE) is the standardized difference between the 2D hydrodynamic model estimates and ADV data.Figure S34.2. Depth and velocity profiles for Plummer Springbrook, at a baseflow of 0.071 cms, as simulated by River2D or obtained with an Acoustic Doppler velocity meter (ADV). Depth transects are displayed above the solid line while velocity transects are below the solid line.


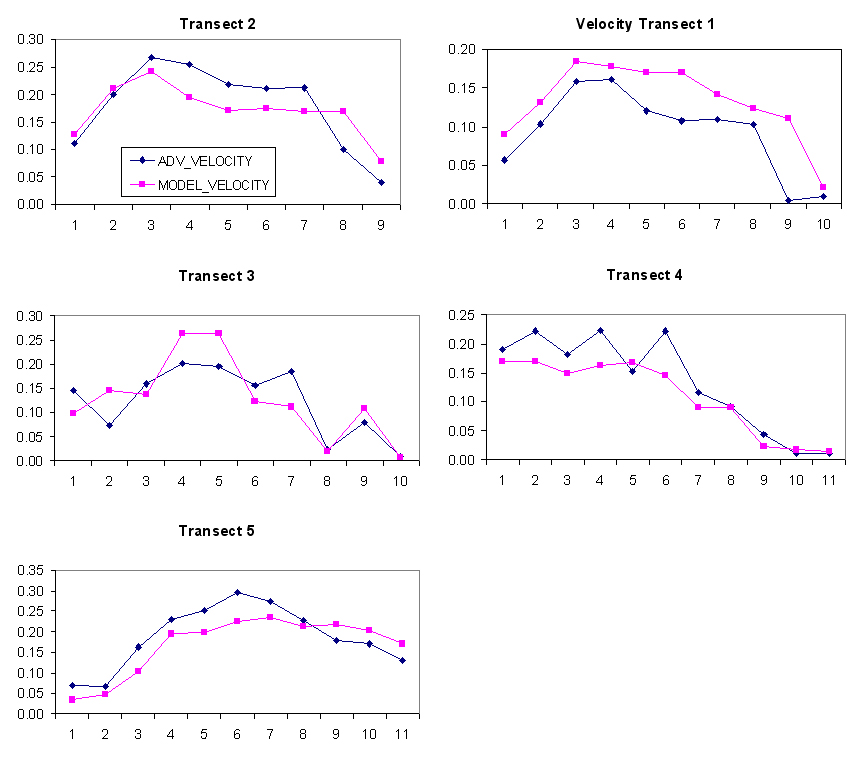


###


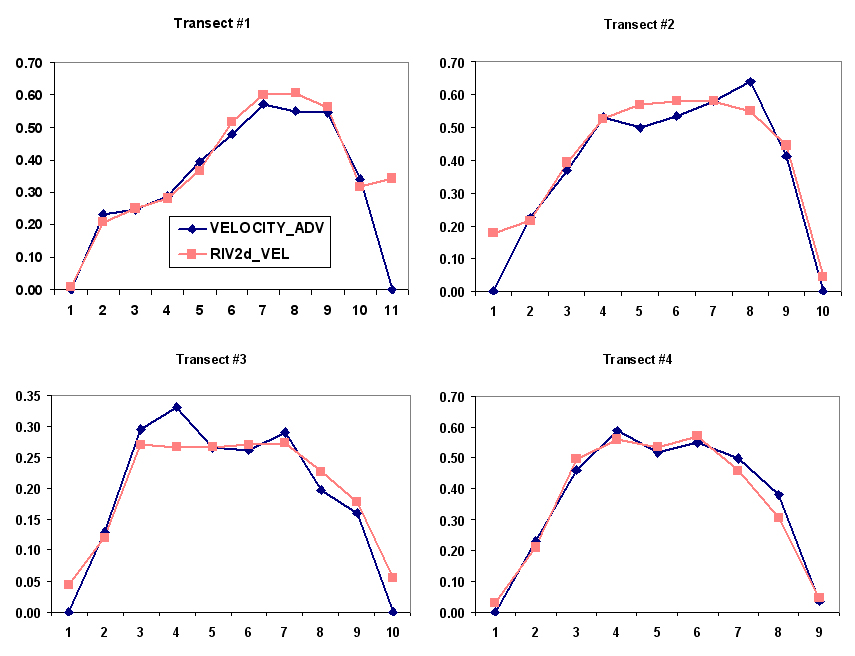


Figure S34.3. Depth and velocity profiles for Pedersen Springbrook, at a baseflow of 0.108 cms, as simulated by River2D or obtained with an Acoustic Doppler meter (ADV). Depth transects are displayed above the solid line while velocity transects are below the solid line.

Pedersen depth (above line) and velocity transect output from the River2D and an acoustic Doppler velocity meter (ADV) for comparison purposes. The root mean square error (RMSE) is the standardized difference between the 2D hydrodynamic model estimates and ADV data.


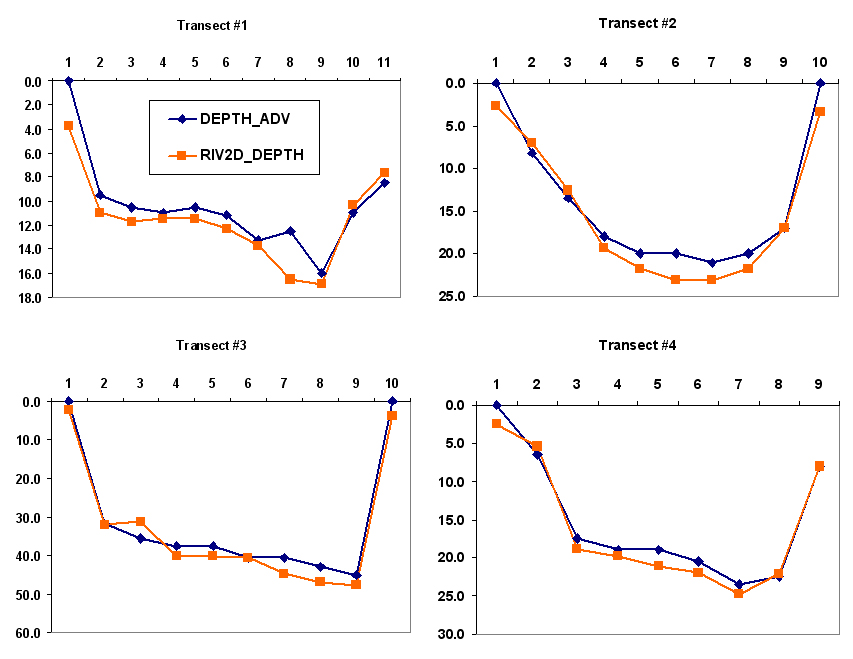

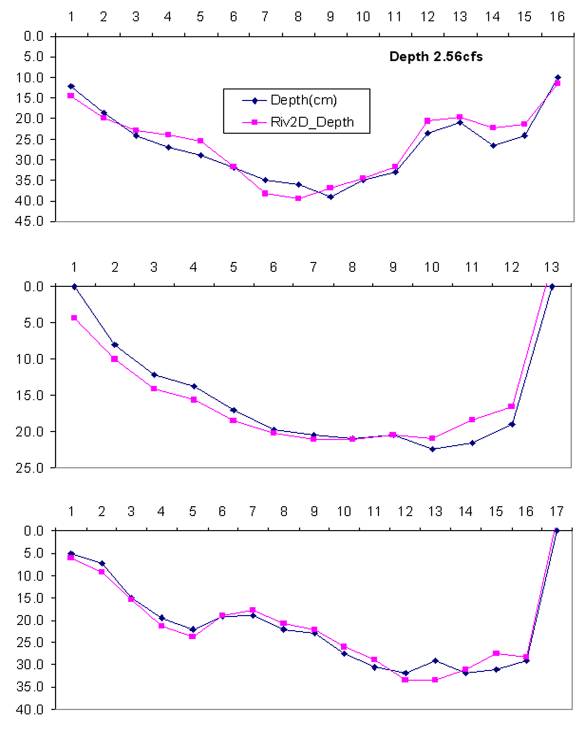

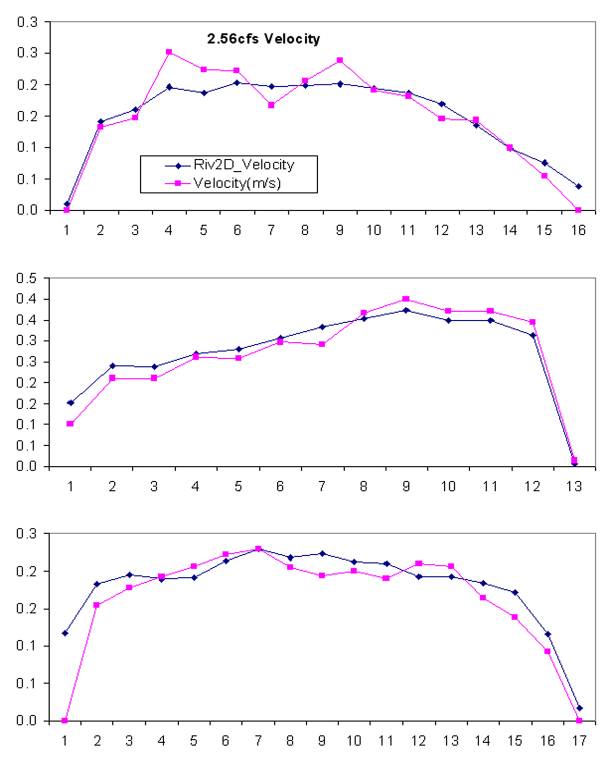


Figure S34.4. Apcar Creek depth (left panel) and velocity (right panel) transects, at a baseflow of 0.066 cms, as simulated byoutput from the Ri River2D or obtained withand an acoustic Doppler velocity metermeter (ADV). for comparison purposes. The root mean square error (RMSE) is the standardized difference between the 2D hydrodynamic model estimates and ADV data.
