# Supplementary material for: An Ecohydraulic Model to Identify and Monitor Moapa Dace Habitat
Source: PLoS One. 2013 Feb 7;8(2):e55551. doi: 10.1371/journal.pone.0055551 (PMC3567127; doi:10.1371/journal.pone.0055551)
Supplement: File S4 — 2D hydrodynamic model output and habitat maps for each springbrook. (DOC) [file pone.0055551.s004.doc]

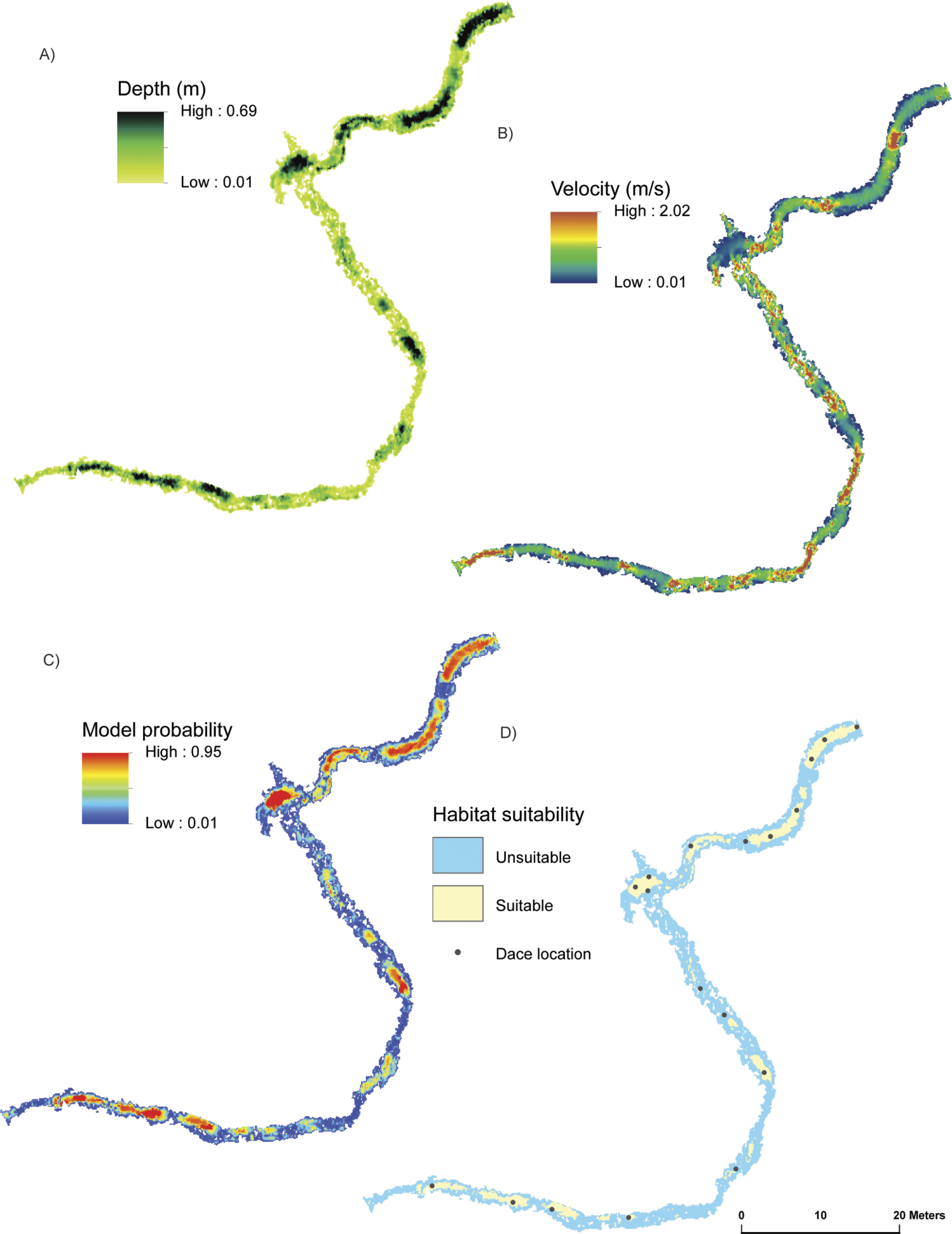


Figure S4.1. Plummer Springbrook depth (A) and velocity (B) simulations output from River2D at a 0.071 cms baseflow, Moapa dace model probabilities (C) and habitat map (D). Dace locations are from a snorkel survey obtained 30 January, 2011.


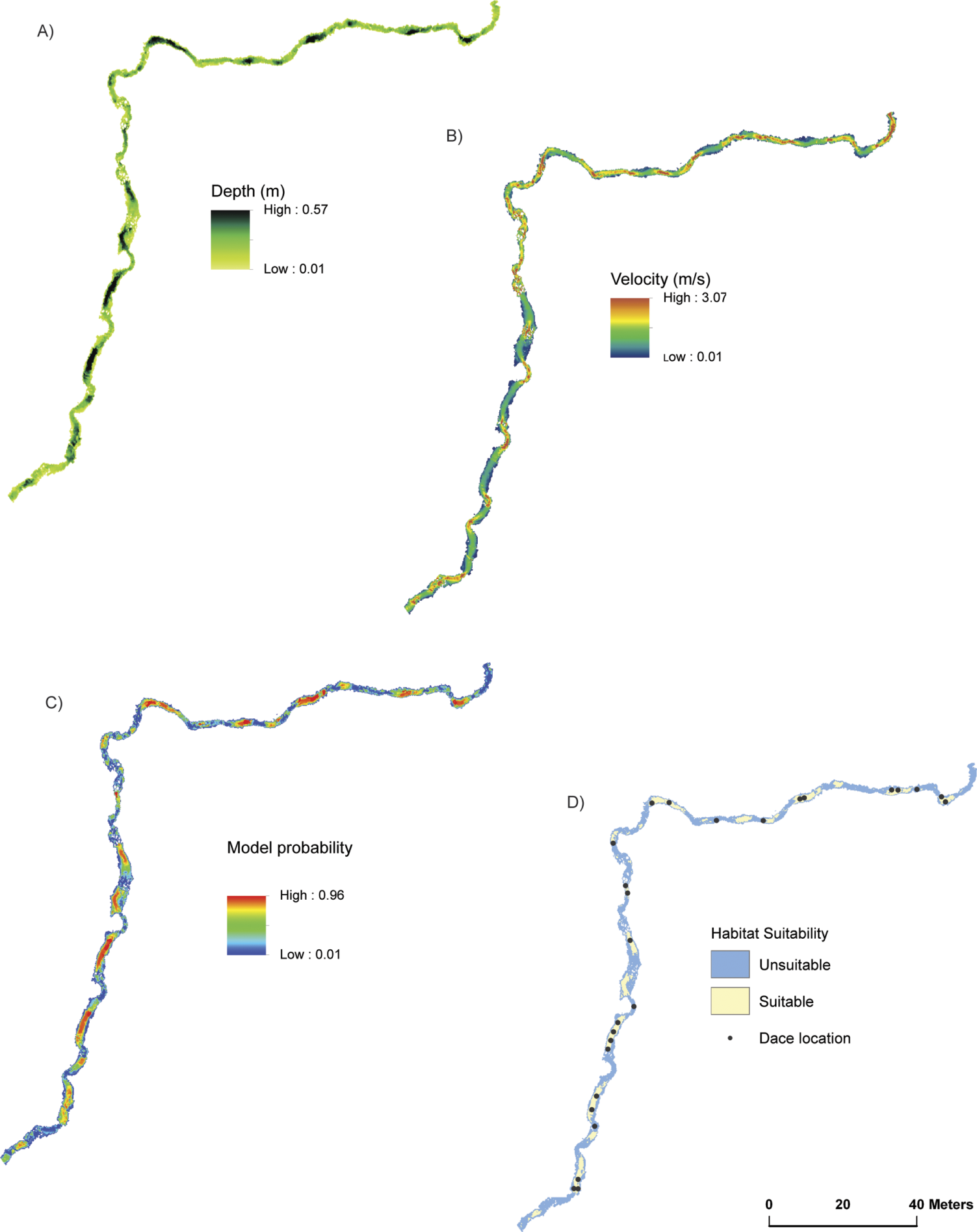


Figure S4.2. Pedersen Springbrook depth (A) and velocity (B) simulations output from River2D at a 0.108 cms baseflow, Moapa dace model probabilities (C) and habitat map (D). Dace locations are from a snorkel survey obtained 30 January, 2011.


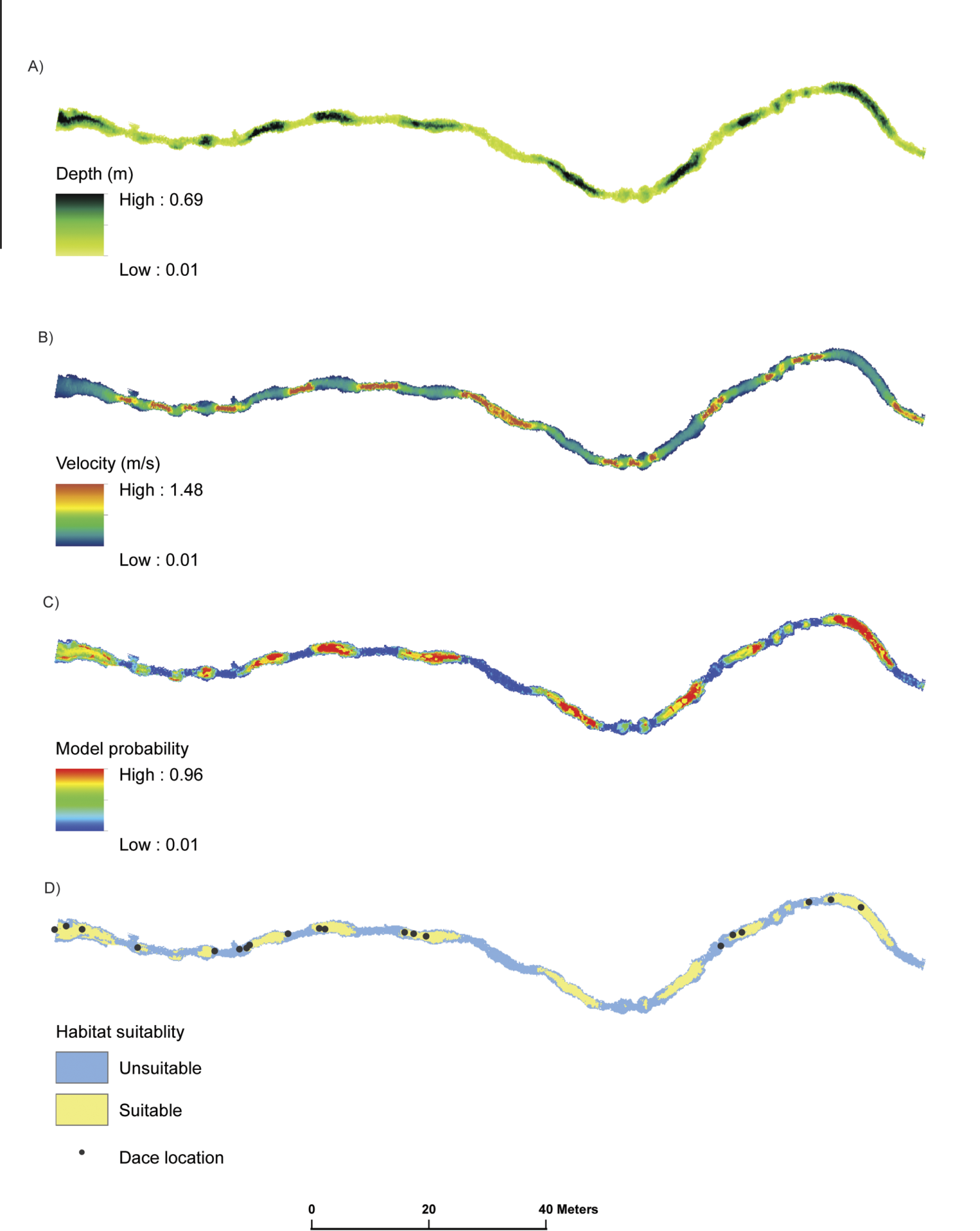


Figure S4.3. Apcar Springbrook depth (A) and velocity (B) simulations output from River2D at a 0.066 cms baseflow, Moapa dace model probabilities (C) and habitat map (D). Dace locations are from a snorkel survey obtained 30 January, 2011.
